# How to treat severe infections in critically ill neutropenic patients?

**DOI:** 10.1186/1471-2334-14-512

**Published:** 2014-11-28

**Authors:** Lara Zafrani, Elie Azoulay

**Affiliations:** AP-HP, Hôpital Saint-Louis, Medical ICU. Groupe de Recherche Respiratoire en Réanimation Onco-Hématologique (Grrr-OH), Paris, France; Medical Intensive Care Unit, Hôpital Saint-Louis, ECSTRA team, Biostatistics and clinical epidemiology, UMR 1153 (center of epidemiology and biostatistic Sorbonne Paris Cité, CRESS), INSERM, Paris Diderot Sorbonne University, Paris, France

## Abstract

Severe infections in neutropenic patient often progress rapidly leading to life-threatening organ dysfunction requiring admission to the Intensive Care Unit. Management strategies include early adequate appropriate empirical antimicrobial, early admission to ICU to avoid any delay in the diagnostic and therapeutic management of organ dysfunction. This review discusses the main clinical situations encountered in critically ill neutropenic patients. Specific diagnostic and therapeutic approaches have been proposed for acute respiratory failure, shock, neutropenic enterocolitis, catheter-related infections, cellulitis and primary bacteriemia. Non anti-infectious agents and recent advances will also be discussed. At present, most of large-scale studies and recommendations in neutropenic patients stem from hematological patients and will need further validation in ICU patients.

**Electronic supplementary material:**

The online version of this article (doi:10.1186/1471-2334-14-512) contains supplementary material, which is available to authorized users.

## Review

### Introduction

Neutropenia is a decrease in circulating neutrophil counts in the peripheral blood. An absolute neutrophil count of 1,000– 1,500 cells/mm^3^ defines mild neutropenia, 500–1,000 cells/mm^3^ defines moderate neutropenia, and <500 cells/mm^3^ defines severe neutropenia. Myelodysplastic syndromes and hematologic malignancies typically cause pancytopenia. A minority of cases present with isolated neutropenia. Moreover, cancer patients may experience neutropenia as a side effect of chemotherapy or radiotherapy. Over the last decades, increased treatment intensity in cancer patients has translated into better survival [1]. More patients are being treated, more intensive regimens are being used, and patients more often undergo stem cell transplantation with the primary goal to control the disease. The result, in most of the cases, is an increase in the number of cases of patients with neutropenia [2]. Infection is the major cause of morbidity and mortality in neutropenic patients [3]. The risk of serious complications depends mainly on the duration of neutropenia (>7 days) and the presence of comorbidities, such as hepatic or renal dysfunction [4, 5]. Infections often progress rapidly leading to hypotension and/or other life-threatening complications requiring admission to the Intensive Care Unit (ICU). ICU admission may be due to inappropriate antibiotherapy. Unfortunately, even when appropriate antibiotics are administrated in a timely manner, neutropenic patients may still end up in an ICU. Indeed, the excessive inflammatory response associated with sepsis may lead to multiple organ failures. In addition, the source of infections is more difficult to identify in neutropenic patients than it is in patients with normal immune function, since symptoms of infection are often diminished. The spectrum of potential pathogens is broad and early diagnosis is essential for guiding treatment and minimizing nonessential drug therapy. In this review we will focus mainly on neutropenia secondary to hematological malignancies and chemotherapy-induced neutropenia in adults.

## Empirical antimicrobial therapy in ICU

In severe infections, empirical antibiotic/antifungal therapy in suspected infections should be tailored to the individual patient to maximize the chances that the therapy is microbiologically appropriate. There is a clear link between microbiologically adequate empirical therapy and successful outcome from infections [6–8].

### Antibacterial drugs

Guidelines have been developed for the management of fever in neutropenic patients with cancer, including hematopoietic cell transplant recipients [4, 9] (Table 1)*.* The Infectious Diseases Working Party of the German Society of Hematology and Oncology published guidelines on the diagnosis and management of sepsis in neutropenic patients where they address specifically the management of critically-ill patients [10]. Unfortunately, prospective randomized studies related to the ICU setting for neutropenic patients are lacking. Therefore, these recommendations are based on studies performed in the non-critically ill patient. The recommended empirical antibiotic therapy is the same as the antibiotic therapy recommended in US guidelines. The aim of empiric therapy is to cover the most likely and most virulent pathogens that may rapidly cause serious or life-threatening infection in neutropenic patients. In all febrile neutropenic patients, empiric broad-spectrum antibacterial therapy should be initiated immediately after blood cultures have been obtained and before any other investigations have been completed [4]. The Infectious Diseases Society of America (IDSA) recommends an empiric monotherapy with an anti-pseudomonal beta-lactam agent, such as piperacillin-tazobactam, cefepime, meropenem, or imipenem [4]. In critically ill patients, combination antibiotic regimens are usually used, although none has been shown to be clearly superior to others or to monotherapy [11, 12]. However most of these data has not analyzed patients who required ICU admission. Such patients remain a subset for which standardized evidence-based recommendations are warranted [13]. Recommended combination regimens include an extended-spectrum beta-lactam combined with an aminoglycoside or a beta-lactam combined with a fluoroquinolone [12]. In the ICU setting, Legrand et al. found that combination antibiotic therapy including an aminoglycoside was associated with lower mortality in neutropenic patients with severe sepsis or septic shock [14]. Vancomycin (or other agents that target gram-positive cocci) is recommended in case of hemodynamic instability, in suspected central venous catheter (CVC)-related infection, in skin or soft tissue infection or severe mucositis and in patients who are colonized with methicillin-resistant S. aureus [4, 15]. Abdominal distension or diarrhea should prompt suspicion of either neutropenic enterocolitis (typhlitis) or Clostridium difficile colitis. Suspected neutropenic enterocolitis should prompt the addition of metronidazole and antifungal therapy for Candida coverage [16].

**Table 1:** **Empiric antibiotic therapy in high risk patients with neutropenic fever (adapted from the IDSA guidelines**[4]**)**

Antibiotherapy	Indications	Grade of recommendation
Antipseudomonal β-lactam agent	All high risk patients with neutropenic fever	A-I
- Carbapenem (meropenem or imipenem-cilastatin)		
Piperacillin-tazobactam		
Aminoglycosides	Hemodynamic instability	B-III
Vancomycin	- Suspected catheter-related infections	A-I
	- Skin or soft-tissue infection	
	- Hemodynamic instability	
Vancomycin, linezolid or daptomycin	Risk of methicillin-resistant Staphylococcus aureus	B-III
Linezolid or daptomycin	Risk of vancomycine-resistant enterococcus	B-III
Carbapenem	Risk of extended-spectrum β-lactamase-producing gram negative bacteria	B-III
Polymyxin-colistin or tigecycline	Risk of Klebsiella pneumonia carbapenemase (KPC)	C-III
- Ciprofloxacin + clindamycin	Penicillin-allergic patients	A-II
- Aztreonam + vancomycin		

### Diagnosis and treatment of fungal disease

The IDSA recommends addition of an empiric antifungal agent after four to seven days in high-risk neutropenic patients who are expected to have a total duration of neutropenia >7 days who have persistent or recurrent fever and in whom reassessment does not yield a cause [4]. However, in ICU patients who are clinically unstable, antifungal therapy should be considered earlier, though no data are available in this specific setting.

The choice of agent for empiric antifungal therapy depends upon which fungi are most likely to be causing infection, as well as the toxicity profiles and cost [4]. In patients who have never been exposed to antifungal agents, Candida spp are the most likely cause of invasive fungal infection. In patients receiving fluconazole prophylaxis, fluconazole-resistant Candida spp (eg, C. glabrata and C. krusei) and invasive mold infections, particularly Aspergillus spp, are the most likely causes [4]. The 2009 North American recommendations propose empirical broad-spectrum treatment with an echinocandin for all ICU patients irrespective of previous exposition to azole agents [17]. This is in accordance with Leroy and al’s study, conducted in neutropenic and non neutropenic ICU patients, that showed a high incidence (38%) of fluconazole resistant Candida, especially in neutropenic patients [18]. Voriconazole or a lipid formulation of amphotericin B are preferred in patients with pulmonary findings suggestive of an invasive mold infection due to higher failure rates with caspofungin in treating invasive aspergillosis, which is the most common cause of mold infections [19]. Of note, large-scale clinical studies on antifungal therapy in immunocompromised patients usually exclude patients with baseline characteristics that are commonly seen in ICU patients, including patients with liver function abnormalities or renal failure or patients requiring vasopressors or mechanical ventilation. In a retrospective study of hematology patients with invasive pulmonary aspergillosis requiring mechanical ventilation in ICU, Burghi et al. found that voriconazole therapy was independently associated with lower mortality [20].

Amphotericin B should be preferred if mucormycosis is suspected, since voriconazole is inactive against mucormycosis [21]. Moroever, in septic patients who have already received an azole antifungal agent, Amphotericin B should be also the drug of choice for empirical therapy.

In addition to gold-standard methods such as blood cultures, histopathology and cultures of fluids or deep tissues, non-culture diagnostic tests may be useful to diagnose promptly fungal disease. Numerous methods have been developed for detecting fungal cell antigens such as Aspergillus galactomannan (GM), 1,3-β-D-glucan or nuclear amplification assays to identify fungal DNA for early noninvasive detection of filamentous fungi in febrile neutropenic patients. A positive (i.e. >0.5 OD) GM test from blood has been accepted as a significant finding indicating a probable invasive fungal infection in severely immunocompromised patients [22]. The 1,3-β-D-glucan assay can be a useful adjunct to blood cultures and biopsy to diagnose invasive candidiasis [23–25]. Some authors suggest that a combination of blood cultures with the β-D-glucan assay or the polymerase chain reaction (PCR) increases the sensitivity of diagnostic testing compared to blood cultures alone [26]. Detection of candida DNA by PCR is more sensitive and provides earlier results for the diagnosis of invasive candidiasis [27]. However, PCR is not yet standardized and so far the value of PCR as early marker of invasive candidiasis remains unclear.

Hepatic or renal dysfunctions should be taken into consideration when choosing an antifungal drug. For example, amphotericin B deoxycholate will be avoided in case of acute kidney injury and voriconazole will be avoided in case of liver dysfunction. In addition, many aspects of antifungal therapy that are relevant to the ICU population have not been sufficiently addressed in clinical studies, including the pharmacokinetic profile of antifungals in patients with underlying renal or hepatic dysfunction; the dose–response relationship; the best route of administration (oral, enteral, or parenteral); especially, drug interactions with frequently used “ICU drugs”.

### Treatment duration and de-escalation of antimicrobial treatment

Guidelines suggest continuing antimicrobial treatment until neutropenia recovery [4]. A recent work by Mokart, D and al. studied de-escalation of antimicrobial treatment in neutropenic patients with severe sepsis [28]. Sixty-eight percent of patients in this serie underwent de-escalation during neutropenia. Authors did not find a deleterious impact of de-escalation on the survival. Of importance, in the de-escalated patients, treatment was never interrupted before neutropenia recovery. Studies about de-escalation are warranted in patients with neutropenia. Indeed, now that it seems feasible, large interventional trials are needed to understand if survival benefits can be expected in these patients [29, 30].

## Clinical presentations and specific management in ICU

**Figure 1:**
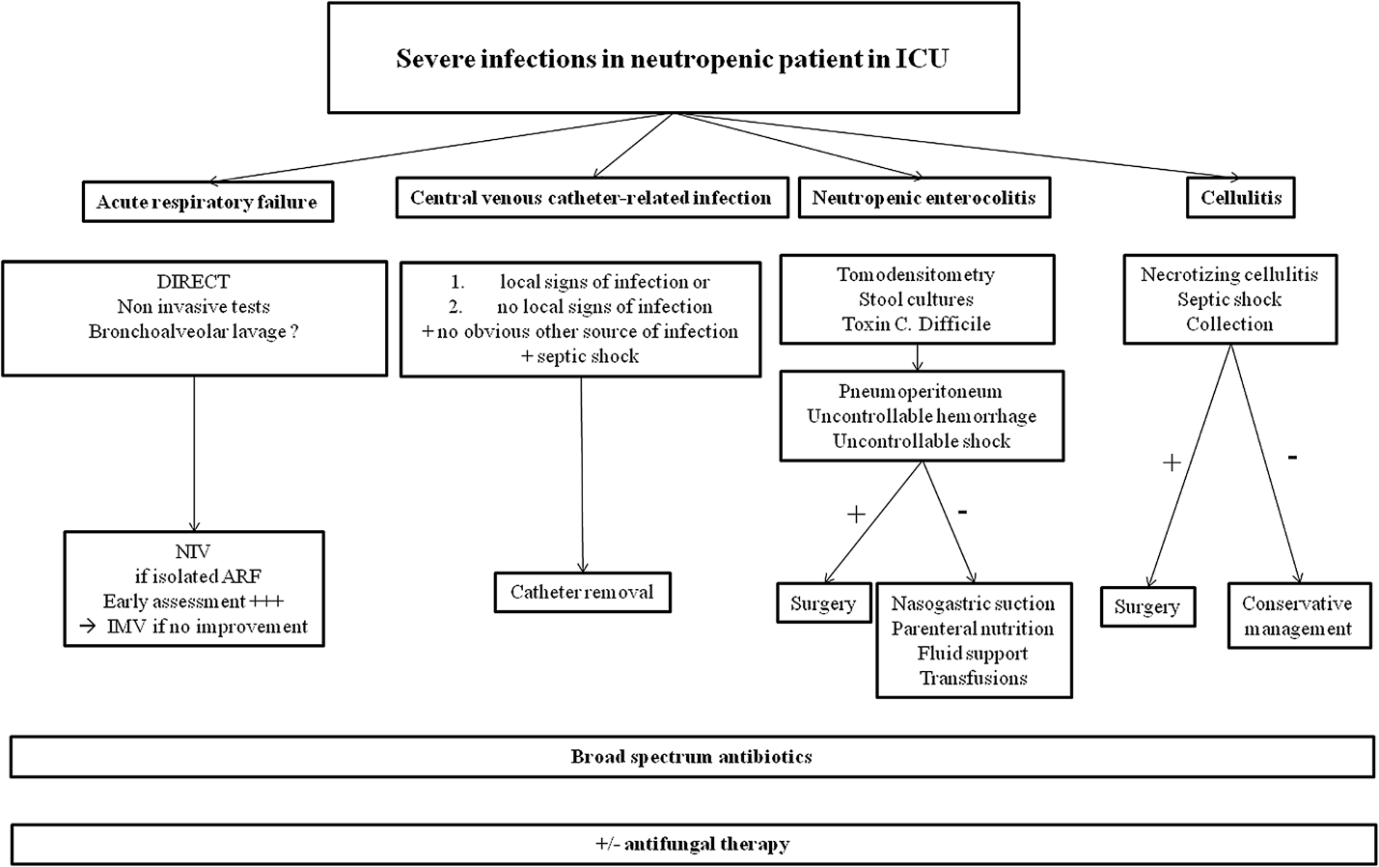
**Management of severe infections in neutropenic patients in ICU.**

Figure 1 summarizes the management of severe infections in neutropenic patients according to their clinical presentation at ICU admission.

### Neutropenia and acute respiratory failure

Acute respiratory failure (ARF) occurs up to 50% of patients with neutropenia [31]. These patients should be admitted early in ICU in order to benefit from early noninvasive diagnostic and therapeutic management. Indeed, the prognosis is worsened when ICU admission is delayed [32]. We described previously the most important criteria to consider when evaluating these patients, using the mnemonic DIRECT [31, 33]: **D**elay since the onset of malignancy or haematopoietic stem-cell transplantation (HSCT), since symptom onset and since the implementation of antibiotics/prophylaxis; pattern of **I**mmune deficiency; **R**adiographic appearance; **E**xperience and knowledge of the literature; **C**linical picture; and findings by high-resolution computed **T**omography (CT) of the chest [31, 33]. The DIRECT method may help physicians to determine the most likely causes of ARF in a cancer patient and guide further investigations and/or empirical therapy.

Fiberoptic bronchoscopy and bronchoalveolar lavage is safe when performed early after ICU admission. However, this procedure adds diagnostic information to that obtained by noninvasive tests in only 18% of patients and had little therapeutic impact. Noninvasive tests identify the cause of acute respiratory failure more frequently and more quickly than does the bronchoalveolar lavage [34]. Conventional chest radiographs show abnormalities in <2% of febrile neutropenic patients without clinical findings indicating lower respiratory tract infection [35, 36]. In patients persistently febrile after >48 h of broad-spectrum antibacterial therapy, 10% of chest radiographs are abnormal, whereas highresolution CT scans at this time reveal pathological findings in 50% of patients [37].

Beyond early identification of lung infiltrates, CT findings may allow for distinguishing fungal from nonfungal lung infiltrates [38, 39]. Nodular or cavitary lesions are suggestive of invasive filamentous fungal infection; however, differential diagnoses include pneumonia due to other microorganisms including mycobacteria, Nocardia, Pneumocystis or Pseudomonas aeruginosa as well as lung involvement by underlying malignancies, so that comparison to previous CT scans in an individual patient is essential [22, 23, 40, 41].

An initial broad-spectrum treatment with combinations of a β-lactam or carbapenem plus an aminoglycoside or antipseudomonal fluoroquinolone is recommended during pneumonia in neutropenic patients [4, 42]. Beside antimicrobial drugs, the question of non-invasive mechanical ventilation (NIV) as opposed to invasive mechanical ventilation in these patients is crucial. Studies evaluating NIV in hematology patients highlight the deleterious effects of NIV failure and late intubation [43–45]. Curative NIV should be discouraged in patients with an associated extra-respiratory organ failure, and in those with mild to severe ARDS. We recommend a cautious use of curative NIV only in patients with isolated ARF and with an early assessment of its efficacy. When no improvement is seen, invasive mechanical ventilation must be considered early to ensure the highest chance of survival for neutropenic patients with hypoxemic ARF. Readers must bear in mind that trials that evaluated NIV at a time where mechanical ventilation was associated with 80-90% mortality are not relevant anymore.

### Acute respiratory distress syndrome (ARDS) and neutropenia recovery

Acute respiratory failure that occurs 3 until days before to 3 days after neutropenia recovery may be associated with a deterioration in oxygenation and exacerbation of pre-existing pulmonary disease [46, 47]. Patients at risk for ARDS during neutropenia recovery are those with pulmonary infiltrates during neutropenia [46]. Other risk factors that have been suggested include delayed or prolonged neutropenia [46], and pneumonia [48]. G-CSF should be avoided in this context (cf infra).

### Catheter removal

Deciding when to remove CVC is a common problem in neutropenic patients in ICU. In patients with bacteremia due to Enterobacteriaceae, enterococci or Pseudomonas, with no local signs of catheter infection or septic shock, microbial growth in peripheral blood before 2 hours after growth in a sample obtained simultaneously from the catheter often indicates bacterial translocation from the intestine [49]. CVC should be considered for removal in patients with septic shock, without an obvious other source of infection as the absence of local signs and symptoms are notoriously insensitive in the neutropenic host. In a cohort study of neutropenic cancer patients admitted to the ICU for severe sepsis or septic shock, Legrand et al. found that routinely removing indwelling catheters early on in patients with no other detectable focus on infection was independently associated with survival [14]. If needed, a new catheter may be placed in a different site.

### Neutropenic enterocolitis (typhlitis)

The intestinal tract is a common site of infection in neutropenic patients. Neutropenic enterocolitis, also known as typhlitis is a life-threatening condition due to inflammatory/hemorrhagic/necrotizing involvement of the lower intestinal tract [50]. Criteria for neutropenic enterocolitis associate presence of fever, abdominal pain and demonstration of the bowel wall thickening of more than 4 mm (transversal scan) over more than 30 mm (longitudinal scan) in any segment by ultrasonography or computed tomography [16]. Moreover, other diagnoses such as *C. difficile* associated colitis, graft-versus host disease, or other abdominal syndromes including cholecystitis, cholangitis, appendicitis need to be ruled out. The management of neutropenic enterocolitis has evolved over the years as clinical experience has grown. Recent studies have reported the success of conservative treatment in most patients. Surgical intervention is now reserved for selected cases of neutropenic enterocolitis based on (1) the persistence of gastrointestinal bleeding despite correction of coagulopathies and thrombocytopenia (2) free air in the intraperitoneal cavity indicative of bowel perforation and (3) clinical deterioration despite optimal medical management. The presence of gaz in the mucous lining of the small or large intestine is indicative of pneumatosis intestinalis. This situation refers to a necrotizing enterocolitis and can be considered as an indication for urgent surgery.

Conservative management is recommended initially when these criteria are absent [50]. Badgwell et al. suggested better outcomes if it was possible to delay surgery until recovery from neutropenia [51]. General supportive measures include bowel rest with nasogastric suction, parenteral nutrition if necessary, and intravenous fluid support. Platelet transfusions may be necessary in patients with severe thrombocytopenia. In our hospital, because of close monitoring needed in these patients, early ICU admission is the rule.

### Perianal cellulitis

Perianal cellulitis should be promptly recognized in neutropenic patients, as they are associated with significant morbidity and mortality [52]. Necrotizing cellulitis and cellulitis-induced septic shock require surgery. However, surgery is sometimes a complex decision and some authors suggest that, in the absence of septic shock, the management depends on the presence of fluctuation or collection [52]. In Morcos et al. serie, patients without collection or fluctuation were treated conservatively with antibiotics [52]. However, patients who are managed conservatively, should be monitored closely by both intensivist and surgeons until they improve. Indeed, sometimes fluctuation/collection or septic shock become evident in the later course.

### Vasopressor regimen during septic shock

International guidelines for management of severe sepsis and septic shock apply for neutropenic patients [13]. Guidelines from the infectious diseases working party of the German Society of Hematology and Oncology [10] for the management of sepsis in neutropenic patient recommend the use of norepinephrine as the drug of choice if a sufficient mean arterial pressure (> 65 mmHg) cannot be achieved by fluid resuscitation, associated with dobutamine in case of sepsis-related myocardial depression . Moreover, D. Schnell and al. studied the impact of a recent chemotherapy on the duration and intensity of the norepinephrine support during septic shock [53]. Cancer patients recently treated with chemotherapy had similar needs in vasopressor support during septic shock compared with untreated cancer patients and patients without malignancy [53].

## Non anti-infectious agents

### G-CSF and GM-CSF

Haemopoietic growth factors, such as granulocyte colony-stimulating factor (G-CSF) and granulocyte-macrophage colony-stimulating factor (GM-CSF) have been assessed in several clinical trials [54, 55]. The known effect of G-CSF and GM-CSF in increasing the number of circulating neutrophil granulocytes was the rationale for clinical studies assessing their role as additional therapy to antibiotics in febrile patients with chemotherapy-induced neutropenia. A meta-analysis of 13 randomized controlled trials showed that G-CSF reduces the time to neutrophil recovery and the length of hospitalization [55]. However, overall mortality appeared not to be influenced. In ICU, in a retrospective study of 28 neutropenic patients who received G-CSF compared to 33 patients who did not received G-CSF, Gruson et al. did not found any difference in terms of clinical outcome and occurrence of nosocomial infections [56]. Moreover, as mentioned above, in patients with pulmonary infiltrates during neutropenia, G-CSF-induced neutropenia recovery carries a risk of respiratory status deterioration with ARDS [57].

### Granulocyte transfusion

Granulocyte transfusions have been most frequently employed in the management of patients with neutropenic sepsis that is unresponsive to conventional antimicrobial therapy. However, therapeutic administration of granulocyte transfusions in the neutropenic host with severe infection has no proven benefit. A cochrane database systematic review concludes that there is inconclusive evidence to support or refute the use of granulocyte transfusions [58]. More recently, in 30 severely ill neutropenic patients with haematological malignancies, Cherif et al. demonstrated a good feasibility of granulocyte transfusions and signs of clinical efficacy [59]. Specific data in ICU setting are not currently available. Moreover, complications of granulocyte transfusions have been reported such as fatal CMV infection, allo-immunization and transfusion-related acute lung injury (TRALI) syndrome. Therefore, granulocyte transfusions are not recommended in routine use and should be avoided in ICU.

### Intravenous immunoglobulin

Polyclonal intravenous immunoglobulins have been suggested to be beneficial during sepsis by modulating the immune response and neutralizing bacterial endo and exotoxins and stimulating serum bactericidal activity. In neutropenic patients with hematological malignancies and severe sepsis or septic shock, the prospective randomized controlled study conducted by Hentrich et al. found no beneficial effect of intravenous IgMA-enriched immunoglobulin therapy [60].

## Emerging trends and recent advances

### Multidrug resistant bacteria

Multidrug-resistant bacteria have become more prevalent among neutropenic patients because of their greater time exposure to the health-care environment and selective pressure from prophylactic and therapeutic antimicrobial drugs. In neutropenic patient, among Gram-negative bacteria, Pseudomonas aeruginosa, Acinetobacter species, Escherichia coli, Klebsielle pneumonia and Stenotrophomonas maltophilia are increasingly found to exhibit multidrug resistance [61, 62]. Antibiotic selection pressure promotes the induction of extended-spectrum chromosomal β-lactamases (ESBL) after the use of β-lactams [63, 64] and the selection of enterobacteria with decreased porin production after the use of carbapenems [62]. Enterobacteriaceae that produce Klebsiella pneumonia carbapenemases (KPCs) that confer resistance to all β-lactams, are now reported worldwide and may require treatment with colistin or tigecycline [65, 66]. Fluoroquinolone exposure is associated with the emergence of methicillin-resistant Staphylococcus aureus (MRSA) and penicillin-resistant streptococci [67]. Vancomycine resistant enterococci (VRE) has become a prominent pathogen in cancer patients. Linezolid and daptomycin have been approved for use in infections with VRE (including those with associated bacteremia) with linezolid often used as a first-line treatment. Early reports of linezolid resistant enterococci occurred predominantly in immunocompromised hosts who resided in an ICU [68]*.*

### New antifungal drugs

– To date, posaconazole is only available in oral suspension. A study evaluating the pharmacokinetics and adverse effects of an intravenous formulation of posaconazole is currently being completed (ClinicalTrials.gov. Pharmacokinetics, safety, and tolerability of intravenous posaconazole solution followed by oral posaconazole suspension in subjects at high risk for invasive fungal infections (NCT01075984). Available from: ClinicalTrials.gov). An intravenous formulation of posaconazole would be of particular interest in ICU patients.

– New antifungal drugs are currently being investigated. However, to date, published data are very scarce. New azole antifungals include Ravuconazole, Isavuconazole and Albaconazole. The spectrum of activity of these 3 drugs, based on in vitro studies, include Candida spp. (including fluconazole-resistant isolates), Aspergillus spp and Cryptococcus neoformans [69, 70]. Ongoing clinical trials involve the treatment of invasive aspergillosis and candidiasis. However, published results are to date unavailable. (Ravuconazole in preventing fungal infections in patients undergoing allogeneic stem cell transplantation 2013; available from http://www.clinicaltrials.gov). Furthermore, to the best of our knowledge, there are no specific study ongoing in the ICU setting. There are also case reports with a combination of a new monoclonal antibody against Candida spp (including fluconazole-resistant species), Efungumab, with other antifungals in invasive candidiasis [71, 72]. However, no large scale studies are published at this time.

### Combination antifungal therapy

Data supporting the routine use of antifungal combinations for invasive candidiasis are limited and controversial [73]. Amphotericin B plus flucytosine is standard therapy for central nervous system (CNS) candidiasis, Candida endophthalmitis and Candida endocarditis. However, clinical data supporting such recommendations are generally lacking [17]. In invasive aspergillosis, the combination of two agents (voriconazole plus echinocandin) is currently recommended only in salvage therapy for pulmonary aspergillosis. However, an unpublished trial has proven no benefit of combination of antifungal therapy. The combination of caspofungin plus voriconazole is also a treatment option for CNS aspergillosis despite the limited CNS penetration of the echinocandin [74].

## Conclusion

Infections in neutropenic patients often progress rapidly and require prompt admission in ICU. Early adequate management, as a part of a collegial medical procedure involving intensivists, hematologists and/or oncologists, includes prompt initiation of antimicrobial therapy and life sustaining therapies. Multidrug-resistant bacteria, particularly from the nosocomial setting, have become more prevalent among neutropenic patients in ICU over the last decades because of their greater time exposure to the health-care environment and selective pressure from prophylactic and therapeutic anti-infective exposure. The development of antimicrobial stewardship program is therefore of utmost importance. To date, benefit of non anti-infectious agents such as G-CSF or intravenous immunoglobulins has not been proven in terms of clinical outcomes. Most of large-scale studies and recommendations in neutropenic patients stem from hematological patients and have to be validated in the ICU setting.

## Authors’ original submitted files for images

Below are the links to the authors’ original submitted files for images. Authors’ original file for figure 1
