# Supplementary figures and images for: How to treat severe infections in critically ill neutropenic patients?
Source: BMC Infect Dis. 2014 Nov 28;14:512. doi: 10.1186/1471-2334-14-512 (PMC4289060; doi:10.1186/1471-2334-14-512)

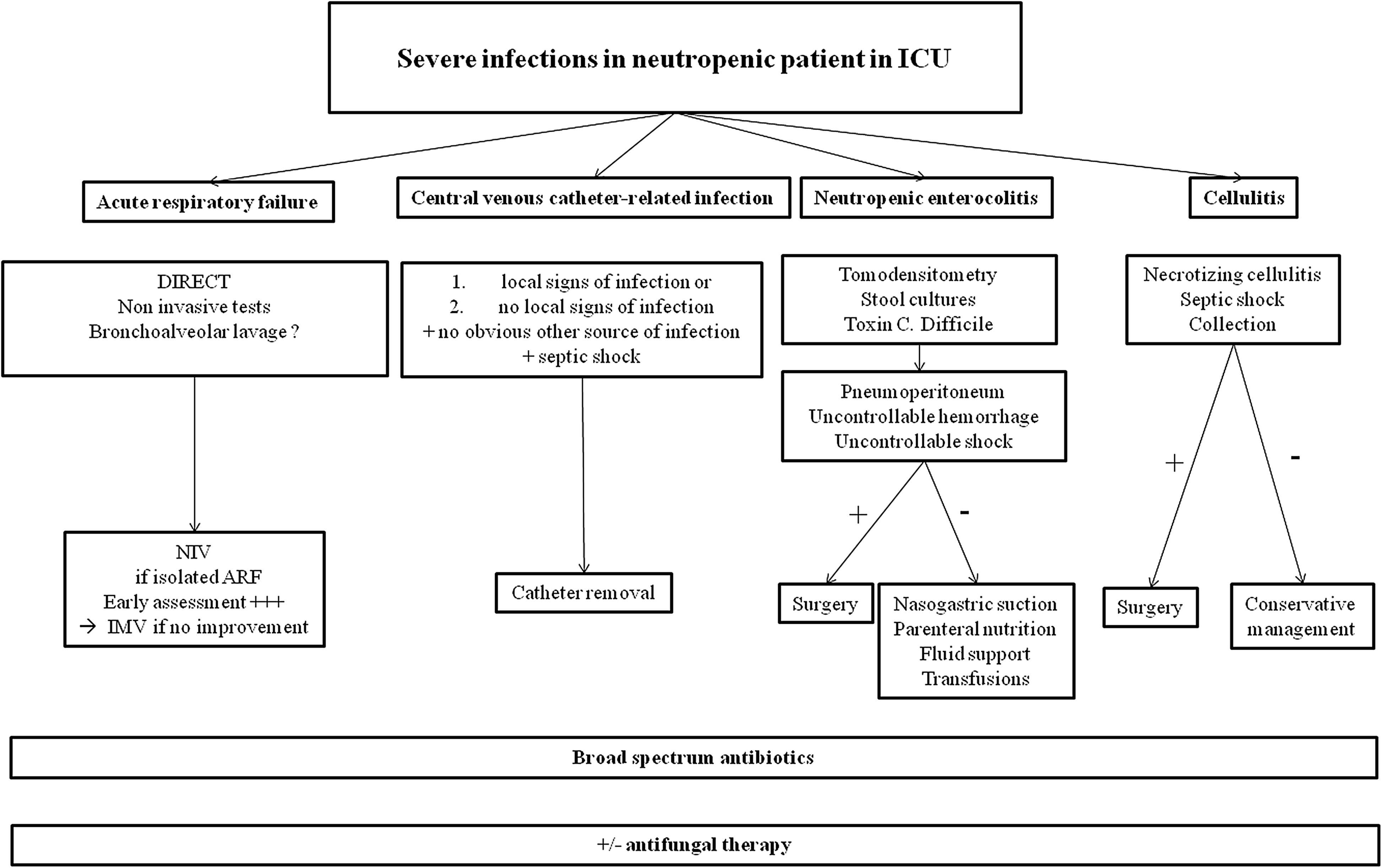

Supplement: Supplementary file 1 — Authors’ original file for figure 1 [file 12879_2014_4066_MOESM1_ESM.tiff]
